# Views and Preferences for Nicotine Products as an Alternative to Smoking: A Focus Group Study of People Living with Mental Disorders

**DOI:** 10.3390/ijerph13111166

**Published:** 2016-11-23

**Authors:** Carla Meurk, Pauline Ford, Ratika Sharma, Lisa Fitzgerald, Coral Gartner

**Affiliations:** 1 Policy and Epidemiology Group, Queensland Centre for Mental Health Research, Locked Bag 500, Archerfield, QLD 4018, Australia; 2 School of Public Health, The University of Queensland, Herston, QLD 4006, Australia; r.ratika@uq.edu.au (R.S.); l.fitzgerald@sph.uq.edu.au (L.F.); c.gartner@uq.edu.au (C.G.); 3 School of Dentistry, The University of Queensland, Herston, QLD 4006, Australia; p.ford1@uq.edu.au; 4 UQ Centre for Clinical Research, The University of Queensland, Herston, QLD 4006, Australia

**Keywords:** nicotine products, tobacco harm reduction, people living with mental illness, qualitative research, consumer preferences

## Abstract

*Aims and Background*: People living with mental disorders experience a disproportionately higher burden of tobacco-related disease than the general population. Long-term substitution with less harmful nicotine products could reduce the tobacco-related harm among this population. This study investigated the views and preferences of people with mental health disorders about different nicotine products and their use as long-term substitutes for cigarettes. *Methods*: Semi-structured focus group discussion followed by a brief questionnaire. The discussion transcripts were analysed for content and themes and quantitative data summarised with descriptive statistics. *Results*: Twenty-nine participants took part in four focus groups. Vaping devices were the most acceptable nicotine products discussed; however preferences for nicotine products were individual and varied along aesthetic, pragmatic, sensory and symbolic dimensions. The concept of tobacco harm reduction was unfamiliar to participants, however they generally agreed with the logic of replacing cigarettes with less harmful nicotine products. Barriers to activating tobacco harm reduction included the symbolism of smoking and quitting; the importance placed on health; the consumer appeal of alternatives; and cost implications. *Discussion and Conclusions*: Engaging this population in tobacco harm reduction options will require communication that challenges black and white thinking (a conceptual framework in which smoking cigarettes or quitting all nicotine are the only legitimate options) as in practice this serves to support the continuance of smoking. Consumers should be encouraged to trial a range of nicotine products to find the most acceptable alternative to smoking that reduces health harms. Providing incentives to switch to nicotine products could help overcome barriers to using less harmful nicotine products among mental health consumers.

## 1. Introduction

The high smoking prevalence among people living with mental disorders is a key contributor to the disproportionate burden of disease they experience [1,2,3,4]. There are several possible reasons why people living with severe mental disorders smoke more than those without mental disorders, including: social determinants of health [5,6]; neurobiological links between addiction and mental illness which cause a shared vulnerability [6,7,8,9]; and possibly therapeutic benefits to smoking such as alleviating symptoms of mental illness and side-effects of psychotropic medications, although this is disputed [8,10,11].

Many people living with mental disorders indicate they would like to quit smoking [12,13]. However, they face significant challenges to accessing appropriate health care such as economic barriers to treatment, and gaps in the delivery of appropriate physical health care by mental health professionals [14,15], including smoking cessation assistance [16]. 

The complex relationships between smoking and mental disorders and the greater challenge this population group faces in achieving abstinence means that including harm reduction options in addition to abstinence-focused goals may be more ethical and effective for these smokers [17]. The concept of tobacco harm reduction has been defined by Kowzlowski and Abrams as the “*acknowledgement of the special deadliness of smoking and development of ways to increase harm reduction in continuing users of lethal tobacco products by displacing smoking with much less harmful tobacco or nicotine*” [18].

A promising tobacco harm reduction approach is to encourage consumers who cannot or do not want to quit using nicotine to switch from cigarette smoking to less harmful alternatives, such as “clean” nicotine products (e.g., patches, gums, sprays or lozenges), the use of smokeless tobacco products, or nicotine vapour products (commonly referred to as e-cigarettes or vaporisers) [19].

### Current Evidence on the Long Term Use of Nicotine Products for Smoking Cessation for People with Mental Disorders

The evidence-base on the efficacy and long-term effectiveness of nicotine products is incomplete for those with mental disorders [6]. There is some evidence that the long-term use of nicotine products in conjunction with psychological therapies or education may be effective in maintaining smoking cessation for people with schizophrenia [20,21,22].

The evidence-base for e-cigarettes, specifically, as an approach for smoking cessation is still emerging and thus subject to change [23]. There has been evidence to suggest that smokers with mental disorders are more likely than those without to have tried or want to use e-cigarettes [24]. However, there is only limited clinical trial data on the efficacy of nicotine vapour products for assisting quitting among smokers with mental disorders. Secondary analysis of data from 86 people with mental disorders in a clinical trial found no statistically significant difference in the efficacy of patches versus nicotine or non-nicotine e-cigarettes for quitting [25]. However, e-cigarette use was linked with a greater reduction in smoking, improved treatment adherence and greater consumer acceptability [25]. A study of 14 smokers with schizophrenia with no intention to quit smoking found that participants who tried an electronic cigarette exhibited a significant reduction in cigarettes per day at 12 months and two participants were abstinent from smoking [26]. Another small study of 21 smokers with serious mental disorders found similar results in terms of reduced use and side-effects while also reporting high levels of consumer satisfaction with e-cigarettes [27].

There is currently very little data on the views of smokers who are living with a mental disorder about tobacco harm reduction and different nicotine products that could be used for this purpose. One extensive qualitative study, that focused on disadvantaged smokers (including those with mental disorders), identified that multiple interpretations of e-cigarettes circulate, including views that could facilitate or impede their usefulness for smoking cessation [28]. The present article sought to examine the preferences of smokers living with a mental disorder for different forms of nicotine products and their views on the use of these products as a long term alternative to smoking. We discuss the implications of our findings for policy, clinical practice and health communication.

## 2. Methods

### 2.1. Sample and Data Collection

Human Research Ethics Approval for this study was granted by The University of Queensland Behavioural and Social Sciences Ethical Review Committee (ethics review number: 2014000093). Four focus groups were held during 2014 and 2015 with clients of community service organisations (CSOs) who provide services for people living with a mental disorder, including those experiencing, or at risk of, homelessness. Focus groups were conducted at the premises of CSOs as part of a larger study on how smoking and smoking cessation was perceived among populations vulnerable to disadvantage [5]. Each participant included in these four focus groups identified as a daily tobacco smoker, was 18 years of age or older and had sufficient English to provide informed consent and participate in a group discussion. Focus group data were audio recorded and transcribed verbatim prior to analysis. 

The discussion followed a semi-structured guide and explored questions about smoking, experiences in quitting, views on available support to quit smoking, different nicotine products, including both therapeutic products and non-therapeutic products, and views on the use of nicotine products as a long term replacement for smoking (Supplementary File). Towards the end of the focus group, participants were shown ten different nicotine products (mouthspray; patch; gum; lozenge; inhalator; dissolvable strips; snus; nicotine aerosol inhaler; e-cigarette; tank style vaporiser) and invited to ask questions. Discussion of products included questions and answers regarding cost and availability.

Following the focus group, a brief survey was administered to elicit demographic information on smoking history, quitting history, participants’ past use of and willingness to try different nicotine products, and questions about quitting medications. Questions asked included: “Have you used nicotine replacement therapy previously (not Champix or Zyban)?” (yes/no); “What types of nicotine replacement therapies have you ever used?” Participants could choose any or all of six nicotine products, available for purchase in Australia, that applied (mouthspray; patch; gum; lozenge; inhalator; dissolvable strips; other; I’ve never used nicotine replacement therapy). Participants were also asked to rate their willingness to try each of ten products (mouthspray; patch; gum; lozenge; inhalator; dissolvable strips; snus; nicotine aerosol inhaler; e-cigarette; tank style vaporiser) on a three-point scale (very likely to try; maybe would try; would never try), which were grouped into two categories (very likely to try or maybe would try = 1; would never try = 2) for analysis.

### 2.2. Analysis

The present analysis combines content and thematic analysis and focusses on participants’ views of nicotine products, including their views on using nicotine products as a long-term replacement for smoking. Carla Meurk read and re-read printed transcripts to summarise participants’ views on the different nicotine products discussed, as well as their views on long term nicotine product use as a substitute for smoking. Relevant extracts were stored in an Excel spreadsheet to help identify patterns in the data. Following initial coding, Carla Meurk circulated summary tables to co-authors (Coral Gartner, Ratika Sharma, Pauline Ford, Lisa Fitzgerald) who were familiar with the data to discuss the validity and utility of initial findings. Discrepancies were discussed and resolved through consultation. Summary statistics on demographics as well as willingness to try the products were calculated using SPSS version 22 [29].

## 3. Results

### 3.1. Participant Characteristics

A total of 29 participants took part in four focus groups conducted in Brisbane and Townsville, Queensland. Participants had a median age of 45 years (range 22–67 years of age), were evenly split by sex (male, *n* = 15 and female, *n* = 14) and three participants identified as being Aboriginal or Torres Strait Islander. The median number of cigarettes smoked per day was 20 (mean = 20.25, SD = 10.02; range 3−50) and under one-half (*n* = 12, 41.4%) of participants indicated that they had tried to quit smoking in the past 12 months.

### 3.2. Experiences and Use of Nicotine Products

As shown in Table 1, more than half (55.2%) of participants indicated that they had used nicotine products previously. Nicotine patches were the nicotine product that most participants had previously used (55.2%).

There were three circumstances in which participants reported that they used, or had used nicotine products: to make a quit attempt; during periods of temporary or enforced abstinence; and as a form of harm reduction. Patches had often been used during in-patient hospital stays to reduce nicotine withdrawal symptoms when participants were unable to smoke.

“Actually, well, I did three days in detox and didn’t have a cigarette while I was in there and I used the patches and that was fuckin’ crazy. […] I was thinking about rolling it up and chewing it and start smoking it, so I couldn’t do that.”

[FG3P1]

A small number of participants described that they used nicotine products along with smoking to reduce their smoking, to act as a stop-gap measure if they didn’t have enough money to buy cigarettes or if they were unable to smoke for some other reason (e.g., while travelling).

“I find a lot of the time I’m buying the quit smoking products other [sic] than cigarettes merely because they might get me through a little bit longer than the smokes will, because I tend to smoke more if I have cigarettes. But in the same point, a lot of the time the price of the cigarettes and the price of the nicotine replacement products are very similar.”

[FG2P1]

One focus group participant, who was also an advocate for the use of vaping devices, reported having switched to a vaping device as a “harm reduction” measure.

“I started on the very small ones, which I think was probably the one that (name) was on, one that looked like a cigarette and they don’t actually give you enough bang for your buck. So for quitting smoking from a 16-a-day smoker, I have moved to … I think it’s like a third stage e-cigarette […] you’re still getting the deep feeling of the nicotine going in, you’re still getting the nicotine, so on a harm reduction it’s been fantastic.”

[FG4P7]

### 3.3. Views on Nicotine Products

A summary of participants’ views on currently available and prospective nicotine products are presented in Table 2.

#### 3.3.1. Vaping Devices

Vaping devices, including tank vaporisers and e-cigarettes, were the most popular nicotine products discussed, in terms of participants’ willingness to try (65.5% willingness in both cases). The focus groups also included a small number of keen advocates for these technologies. Consequently, the topic of vaping devices generated the most discussion.

Reported benefits of vaping devices included that they replicated the psychological and sensory experience of cigarette smoking (visible vapour, taste and smell). Vaping devices were also viewed as having fewer negative effects on others, such as the absence of the smell of smoke.

“It looks like smoke coming out and it doesn’t taste that bad. It’s sort of like you’re getting … because you can see the smoke and it is psychological in the brain.”

[FG1P3]

Personal preferences were evident in views on different kinds of vaping devices. While most participants liked the e-cigarette because it closely mimicked the positive elements of smoking cigarettes without its negatives, others indicated that e-cigarettes were an unattractive simulacrum and in some sense juvenile. For example, some compared them to a type of popular confectionary that resembled cigarettes that were once marketed to children.

“When you took a drag it would look like those lollies you bought that used to have the red stuff on them.”

[FG3P2]

There were related divergences in views on the tank vaporisers. Consistent with the idea that e-cigarettes were juvenile, some liked that the tank vaporisers looked different to cigarettes and were more clearly an adult product. Others took a contrasting view, identifying that the visual dissimilarity to cigarettes was a negative feature and a barrier to use, with some even pronouncing them to be “*ridiculous*” [FG4P1].

“The thing is they’re not trying to look like a cigarette. They are clearly something different. You can personalise them and they come in different colours. You can get some that are a bit quirky. They treat you like an adult with something you might want.”

[FG4P7]

Another respondent’s comment

“It doesn’t look like a cigarette should, would not make me want to smoke it.”

[FG1P4]

The relative size and weight of different vaping devices were also brought up as practical considerations. Those who liked e-cigarettes described them as “*compact and […] seems easy to use*” [FG4P9]. In contrast, tank vaporisers were viewed, negatively, as “*bulky so it’s not going to fit in like your shirt pocket or your jeans or something.*” [FG1P7]

One participant with experience of vaping devices, identified that they had transitioned from e-cigarettes to a tank vaporiser because the latter provided a more satisfying “hit”.

“I think just from experience I think you start off on the smaller one [e-cigarettes] because the ones that are like a cigarette, but as a smoker you’ll find they don’t quite give you enough and that is probably where you progress [to a nicotine vaporiser], it’s more like going for a larger tube. So it’s a bit of a culture around that. I think it’s about having more than one so people can go onto them.”

[FG1P7]

Given the lack of widespread availability in Australia of nicotine solution for refilling the devices, the need for organisational skills was raised as a necessary requirement for successfully switching from cigarettes to vaping devices. Some participants also reported concerns regarding the quality of nicotine liquids available.

“I think you would have to be organised and organise your finances and make sure that when it does run out you’ve got something to fill it up with, because that would be the time when you go, “Oh bugger I’ve run out of this” and you would go and buy a packet of cigarettes or whatever. Do you know what I mean?”

[FG1P5]

While generally positive regarding the prospect of vaping devices, some concerns were raised by participants about possible negative health effects as well as cost, environmental impacts and legal issues.

One participant reported that e-cigarettes had exacerbated their asthmatic symptoms.

“I found a problem with them and I tried them for a while and I get a bit of asthma and I found with the vapour it would make my lungs rattle a bit, so I would worry that long term you might get pleurisy or something from taking in the moisture, a bit of fluid on the lungs”.

[FG1P7]

Another participant did not like the idea of the environmental impacts associated with the disposable e-cigarettes, describing them as “*pretty environmentally unsound*” [FG3P4].

Some participants foreshadowed possible legal issues with the tank vaporiser, if it was mistaken for a “*drug implement*” by police [FG1P5] and it was recognised that these vaporisers could be used to consume cannabis oil.

Initial outlay and ongoing costs were a topic of discussion among participants and it was generally agreed that vaping would need to cost less than smoking to be an attractive option. Finally, vaping devices were not considered acceptable by those who expressed their desire to quit the “habit” of smoking and transition completely away from a lifestyle that included smoking.

“The cigarette thing I would not be interested in because all you’re doing is you’re still doing the same thing, basically […]. You’ve got to get away from that mindset of having to have something in your hand, you know.”

[FG1P6]

#### 3.3.2. Nicotine Aerosol Inhaler

The Nicotine Aerosol Inhaler was the third most popular nicotine product discussed (58.6% willing to try). Like e-cigarettes, many viewed the nicotine aerosol inhaler favourably for the way that they mimicked the look of cigarettes and the behaviour of smoking.

“That would be alright and I could sit there with that in my hand I’d be alright. […] and it looks like a packet of cigarettes […] I like that you’re still holding the smoke.”

[FG1P5]

However, some participants were not interested in the aerosol inhaler for that very reason—describing that they wanted to give up the habit of smoking rather than simply substituting it with a similar behavior.

“Everybody else thinks it’s the smoking, I’m not really sure that […] Yeah but I’m not really sure that is why I smoke so having something like that really doesn’t attract me at all. […] [I want to get] rid of the habit.”

[FG3P3]

Once again cost was raised as a factor. Participants highlighted that products that were presented as alternatives to cigarette smoking needed to be cheaper in order to incentivise their use.

“I’d put it in the Quit thing or whatever. I don’t know, I think people would use it if it was financially viable. But if you’re having to give up things, [for] the same price as the cigarettes then the smoker is going to go for the cigarettes every time.”

[FG2P3]

The issue of generating product appeal was raised by one participant who highlighted the importance of making the product a desirable, rather than consoling, alternative to smoking. Comparison to a popular mid-strength beer (XXXX Gold) was given as an example.

“If it’s marketed as something cool party people want to use or that kind of thing I reckon it could work but that’s it. If it’s associated with losing something or giving something up then it’s got more nostalgia attached to it but if it’s seen as the new thing and everybody is doing this, have your XXXX Gold and your aerosol nicotine inhaler.”

[FG1P7]

#### 3.3.3. Mouth Spray

Most participants were unfamiliar with the nicotine mouth spray but more than half expressed willingness to try it (55.2%). However, the one participant who discussed having tried the product described a negative experience in which they used too much, resulting in nausea. One participant expressed scepticism regarding the potential effectiveness of the mouth spray, due to its mode of delivery.

“I tend to think that because it’s just something that you spray in your mouth or whatever you don’t have the same effect of picking up a cigarette and putting it in your mouth like ordinary cigarette that might have a better chance of working.”

[FG1P4]

Taste was deemed an important factor in assessing the acceptability of this product by those who had not tried it before.

#### 3.3.4. Lozenge

The participants with experience using nicotine lozenges seemed evenly split as to whether they thought lozenges had been effective or not. Just under one-half of participants (48.3%) expressed their willingness to try this product.

One participant commented positively regarding the similar kinetics of lozenges relative to smoking, albeit when used in tandem with another nicotine product:

“Yeah, I’ve tried the lozenges and I went for about five weeks without smoking in conjunction with an e-cigarette that didn’t have nicotine in it at that stage. I couldn’t find any that did, but I went for five weeks on the lozenges and that was quite good, and it was a bit of a distraction having them in your mouth too, you can sort of roll them around. […] so I still get the feeling of having a cigarette.”

[FG1P7]

Participants showed distinct preferences for lozenge type, including size and flavour, and had divergent opinions regarding their cost-effectiveness. One participant discussed feeling that nicotine lozenges were more addictive than cigarettes.

“I’ve tried the Nicabate [lozenges] which is now off the market. […] They’re off the market now and they were really good but they were very expensive and very addictive. I found that there was something, I was saying maybe there was something in them that Australia wouldn’t allow and so they took it off the market. It just, it would really give you a fix. [...] Yeah, and it was too full on because I needed them more than I needed a cigarette ... really badly. It was terrible.”

[FG2P1]

Other criticisms of lozenges included disliking their taste and finding them ineffective at alleviating cravings.

#### 3.3.5. Inhalator

Participants had mixed views on the inhalator, with slightly more participants expressing negative views than positive ones within the context of the focus group. Fewer than one-half (44.8%) of participants indicated a willingness to try inhalators. Positive aspects of the inhalator that were identified included that it mimicked elements of smoking, although some participants considered inhalators to be less appealing than the vaporising devices because the inhalators did not produce a visible “smoke”.

“I don’t mind those, it gives me the drawing, […] It gives you that feeling of smoking, that sensation which is something to do with your hands side of things and if you are sitting around a bunch of smokers or you’re in a situation then you’ve got something that’s not quite, that’s similar to what having a cigarette might be tempted to be able to do instead of it, yeah.”

[FG1P2]

Another respondent’s comment

“No good […] because the vapour you see the smoke coming out and you’re drawing on something, the vapour is going to work.”

[FG1P1]

One person who had tried an inhalator commented that they appreciated that it had no discernible taste, while another who had a damaged throat described the product as “*burn(ing) my throat*” [FG4P4].

A considerable downside was the visual appearance of this product. Many described the product as looking like a tampon, with one participant commenting that they were embarrassed to be seen using it in public.

“You do feel crap using those. They are alright to use at home but they look so ridiculous when you’re outside.”

[FG4P7]

One participant’s account of inhalator use, highlighted a common theme in participants’ accounts of poly-nicotine product use. This participant described themselves as having “*OD’d*” [FG2P1] from using the inhalator in conjunction with a patch. Participants frequently reported combining the use of different nicotine products, as well as using nicotine products while continuing to smoke cigarettes. Some described experiencing nausea while using multiple products, possibly due to over-use.

#### 3.3.6. Strips

Dissolvable oral strips were mostly unknown to participants and tied with the inhalator in terms of acceptability (44.8% willing to try this product). The two participants who reported having tried dissolvable strips did not consider them to be effective, with one participant stating that they did not find the strips “*strong enough*” [FG2P1].

#### 3.3.7. Snus

Snus, patches and gum were tied in last place in terms of participants’ willingness to try and fewer than half (41.4%) expressed a willingness to try these products. Focus group participants were mostly unfamiliar with Snus, a smokeless tobacco that is not available for sale in Australia. Consequently, participants expressed uncertainty and mixed interest, with some proclaiming that “*it looks weird*” [FG3P1].

In the context of discussing an unfamiliar product, many participants identified the need to be able to “*try it first to find out if it’s for them*” [FG2P2]. Participants highlighted that different options, including snus, might work for different people, depending on their preferences.

“Might take off, I don’t know about other smokers, but for a person that likes the draw, no, but for other people, yes, because other people like the chewing gum or it works for them, so it possibly could.”

[FG2P3]

Some also pointed out that trialling of nicotine products needed to be subsidised as they could not afford to try multiple products in order to find the best option for them.

Snus was recognised as having fewer health harms than cigarette smoking as well as having the advantage of being able to be consumed in areas where smoking was banned. Barriers to using snus included its being an unsuitable option for those with oral prostheses.

“You can use it on the bus, health concerns. You’re not dragging tar into your lungs.”

[FG1P7]

#### 3.3.8. Patch

As noted already, although one of the least favoured, patches were the most commonly experienced nicotine product discussed. Many participants had used nicotine patches during periods of hospitalisation in a smoke-free facility.

“When I was there it lasted the whole five days and I didn’t have a craving whatsoever.”

[FG3P2]

While some positive experiences were mentioned, most of the discussion centred on negative experiences with the product, particularly side effects such as nightmares and allergic reactions.

“If you ask me the patches all they do is give you nightmares, if you put them on while you are asleep you get nightmares.”

[FG1P1]

Another respondent’s comment

“I think I must have been allergic to the adhesive because I would pull the damn thing off and there would be these really itchy red, angry red you know like a rash.”

[FG1P3]

Some participants also found them ineffective, particularly insofar as they didn’t replace the behavioural aspects of smoking.

“The patches don’t do anything because they aren’t addressing that to do with your hand and the psychological…”

[FG2P3]

#### 3.3.9. Gum

Views on chewing gum were mixed, although mostly negative experiences were reported. One participant reported that they were currently using gum and that it was working for them [FG2P2]. However, others described it as unpalatable and difficult to use as directed.

“The chewing gum is useless. […] I’ve tried once the gum and like you said it’s like licking an ashtray. It is absolutely putrid.”

[FG1P3]

Another respondent’s comment

“You are supposed to chew it and then park it. But I didn’t know that when I’d done it too so it was 15 s it was like hard. […] No, disgusting. I wouldn’t recommend it to somebody.”

[FG3P1]

Similarly to the Snus, participants with oral or dental prostheses reported that they could not use the gum.

### 3.4. Views on the Use of Nicotine Products as a Long Term Substitution for Smoking

Participants wavered in their views on the idea of using nicotine products as a long term substitute for smoking. When asked directly about their interest in long-term substitution of smoking with nicotine products, participants generally responded in the affirmative, that they would be interested in substituting smoking for a less harmful alternative. However, their deliberations on this topic indicated a more complex narrative and several impediments to activating this disposition were identified (Table 3).

#### 3.4.1. Swapping One Addiction for Another

Some participants were wary of the idea of trying to substitute smoking for another “habit” because they did not differentiate between different kinds of habit, which they viewed globally to be an undesirable compensating mechanism.

“It would be like swapping smoking to take up gambling or something. It’s about, it’s about… being able to condition yourself into not requiring anything as a crutch.”

[FG2P1]

Another respondent’s comment

“[…] long term habit? No. […] You may as well just keep smoking if it’s just going to be another habit”.

[FG2P3]

This discourse aligned with moral conceptualisations of addiction and in some cases participants indicated that the habitual behaviour of smoking was more concerning than the health harms it caused. However, the concern with addiction itself also resonated with participants’ recounting of how their use of different addictive substances was linked. This included highlighting that cutting down or quitting smoking could promote an increased use of other (harmful) substances and vice versa.

“I haven’t had a drink for three months now it has been for me, but I find I’m using these cigarettes to substitute the alcohol more.”

[FG1P6]

#### 3.4.2. What It Means to Quit

Another reason for not liking the idea of switching to a long term nicotine substitute was that it was inconsistent with their pre-existing conceptualisation of stopping smoking, which involved an idealisation of radical change or transformation in their lifestyle. Those who viewed the idea of quitting smoking as transformational valorised the idea of becoming a non-smoker. Continuing to use nicotine in another form conflicted with this ideal.

“If you’re going to quit smoking then you’re quitting a lifestyle [later in the interview] I think what would work for me [to quit] is going to a day spa for two days and getting completely cleansed internally and externally and then coming out like a butterfly.”

[FG2P4]

In a similar vein, several participants highlighted how diet, and eating healthily, was an important aspect of quitting smoking. 

#### 3.4.3. Health Risks

Overall, participants expressed mixed views regarding the extent to which product health risks would influence their smoking behaviour with regard to switching or quitting. A number of participants pointed out that they continued to smoke in spite of knowing the health risks associated with it.

“I love a cigarette. If I can’t have one in the morning when I wake up then I just go back to bed. I’m starting to feel like that often now too, so … but yeah. No I would be happy to die of lung cancer.”

[FG3P1]

Some acknowledged that observing other smokers with tobacco related disease might be insufficient reason for them to quit but that they would quit if, and when, they discovered they were ill.

“I recently just found out a very close friend of mine […] got lung cancer and […] tumours on the brain. […] it hasn’t stopped me smoking, but every time I have a smoke I think about it. […] to be honest I don’t want to quit at the moment, and I think it will probably take something like where you’ve got cancer for you to quit.”

[FG1P5]

One participant believed that the chemicals in cigarettes, in addition to nicotine, were part of what made them enjoyable, suggesting that a clean nicotine product would be unable to provide an adequate replacement.

“Because it’s phosphorous, like 43 different chemicals to make it taste better.”

[FG1P8]

There was some indication that at least some smokers were applying a higher standard to their deliberations over the health risks of alternatives to cigarette smoking than to cigarette smoking itself, demanding that any alternative must be completely risk free to consider using it.

“If it had no health risks to it, yes. […] I wouldn’t be interested if it was a pill and if it was going to affect my heart, my liver, my kidneys then I wouldn’t be interested at all.”

[FG2P3]

#### 3.4.4. Appeal

The need for an alternative to smoking to be viewed as an attractive consumer item was identified by some smokers. Along with a prominent (although not universally shared) interest in using alternatives to cigarette smoking that shared its aesthetic and sensory elements without its negatives (e.g., smell), one participant identified that associating nicotine products with the loss of something made it a less appealing option than if it was positively marketed as a desirable lifestyle accoutrement.

“I think that not every nicotine product needs to be marketed as a quit aid. I think some nicotine products can be marketed as part of a lifestyle …”

[FG2P4]

#### 3.4.5. Cost

Several financial angles were discussed with respect to the idea of substituting smoking for other nicotine products. Firstly, was the cost of smoking relative to the cost of nicotine products. The (increasing) cost of smoking was an important driver to quit. Relatedly, however, the idea of substituting smoking for an alternative that could prove just as costly, made this switch unappealing.

“When you say “long term” the objective is to give up isn’t it? You wouldn’t want to be smoking them [inhalator] for the rest of your life would you? […] I’m thinking of the expense which is the big drawback for smoking.”

[FG1P4]

As discussed earlier in reference to specific nicotine products, the second cost issue that was discussed related to the perceived need to incentivise smokers for the presumed inevitable trade-offs involved with changing from cigarettes to other nicotine products. Unless the alternative was just as satisfying as smoking, participants recognised that it would be hard to maintain the alternative habit. Accordingly, there was a strong belief that there should be government subsidies for less harmful nicotine products. In the same manner as deliberations over health risks, it appeared that the cost-effectiveness of alternatives to smoking were judged according to a higher standard than that of cigarettes.

The final cost issue that participants raised pertained to the opportunity cost of trialling different types of products in order to find the one that was most acceptable. Participants’ preferences varied between individuals and consequently the number of products a smoker may need to trial in order to find one that worked represented a high potential cost.

“Well we don’t earn a $1,000 a week so we can’t afford to get something unless you’re absolutely 100% it works. We haven’t got time for gimmicks. […] I can’t even go to McDonalds to buy take-away, let alone buy something that is $38.”

[FG1P1]

## 4. Discussion

### 4.1. Implications for Communication

Preferences for nicotine products were very individual and varied along differently regarded aesthetic, pragmatic, sensory and symbolic dimensions. Tobacco harm reduction in the form of long-term substitution of cigarettes with a less harmful nicotine product was a foreign concept to most participants, with many indicating a belief that quitting smoking was an “all or nothing” activity. This finding corroborates similar studies showing a pervasive belief among general population smokers that unassisted quitting is superior to assisted quitting and negative views of the idea of using medications to aid quitting [30,31,32]. Nevertheless, participants generally indicated an understanding of the benefits of using a less harmful alternative to smoking when it was put to them. 

The findings indicate the need to develop resources tailored to this population group that describe the concept of tobacco harm reduction in a relatable way. Educating consumers is important, however, accurately communicating the relative risks and benefits of less harmful nicotine products compared to smoking and also complete abstinence is more complex than the simple “quit” message. Furthermore, health promotion materials should not over-emphasise the health harms of smoking as a reason to switch because we found this message may be met with apathy by smokers, who are aware that they have continued to smoke in spite of knowledge of its negative consequences. Instead, focusing on the benefits of switching may be more appealing to this group. There are obvious ethical downsides to marketing alternatives to smoking if it makes these products appealing to current non-smokers. Consequently, communicators face an important challenge in balancing the need to promote the benefits of a less harmful alternative to smoking, without relying on health arguments, while at the same time not making alternative nicotine products an appealing consumer or lifestyle product to non-smokers. 

### 4.2. Implications for Behaviour Change

At an individual level preferences for nicotine products were divergent. While further research is needed to confirm and extend the findings presented here, identifying which elements of smoking (i.e., aesthetic, pragmatic, sensory or symbolic values) appeal to smokers might help practitioners identify the nicotine product that will be most suitable to their clients. Additionally, there is a need for health professionals to challenge black and white thinking around smoking and quitting that serves to support smoking behaviour. Even among those who appeared interested in quitting smoking, there was a tendency to overemphasize the negative health, financial, effectiveness and lifestyle elements of alternatives to smoking in their deliberations [33]. While an idealisation of becoming a healthful and thrifty non-smoker appears to be part of what motivates some participants to want to quit, it also works against those who may struggle to quit smoking without assistance and/or may not be able to quit nicotine. 

There was some indication that side-effects, including possible initial over-use of nicotine products may form a barrier to continued use for some participants. Advising smokers from this population group on how to use nicotine products correctly, therefore, may need to address overuse in addition to the more common problem of under-use [34,35]. 

### 4.3. Policy and Practice Implications

There are two key policy and practice implications for this study. The first pertains to the costs of trialling products. Our focus group highlights the individual nature of preferences for nicotine products, and the likelihood that finding the right nicotine product for an individual could be outside the financial means of those with limited financial resources. Patches were the most commonly experienced nicotine product discussed. This is likely a direct reflection of their widespread use in in-patient settings, and subsidisation on prescription in Australia. Yet, patches were among the least preferred nicotine products, in terms of willingness to try. Despite smoking cessation guidelines recommending combination therapy (e.g., patches + quick release nicotine products such as gum, spray etc.) [36,37] for smoking cessation, current PBS subsidies are only available for nicotine patches and other cessation medications when prescribed by a doctor. Our study highlights the case for providing subsidies for a wider range of nicotine products so that participants are enabled to find the right combination for them. In the absence of changes at a national level, CSOs and local health organisations may like to consider how they can subsidise their consumers to enable trialing a wider range of nicotine products (e.g., by providing trial-packs that include a range of nicotine products). Nicotine products are currently already less expensive on a daily basis than cigarettes, which was noted by some participants. More emphasis on the financial benefits of switching could be more effective in disadvantaged populations who continue to smoke despite having very limited resources. Economic arguments should take into account the fact that short-term rather than long-term economic concerns may be the strongest driver of economically-driven decision making among this group as financial hardship and housing insecurity could place limitations on one’s ability to maximise savings (e.g., limitations in the ability to capitalise on bulk-buy discounts).

These focus groups were conducted in Australia, where approved medicinal products are the only nicotine products that are permitted to be sold or possessed, apart from smoked tobacco products [38]. Despite these legal restrictions some had experience of vaping nicotine. It was also notable that despite these restrictions and generally low prevalence of use in Australia, more participants were interested in trying nicotine vapour products than any other nicotine product. The unique attributes of these products, most notably the visible “vapour”, may make these products a more attractive option to smokers in this population group than other nicotine products, even in the absence of widespread use in the community. Alternatively, the fact that these products are not widely available—their novelty—may have been what made them interesting to participants. Current laws are a substantial structural barrier to non-therapeutic alternative nicotine products, such as vaping nicotine or using snus. Medicinal nicotine products may be the only viable alternatives for many smokers from disadvantaged backgrounds. As identified by some participants, prolonged use of non-therapeutic nicotine products (generally purchased from overseas via the internet) requires a high degree of organisation making it infeasible for many. In the absence of wider access to these alternative nicotine products, consideration should be given to allowing interested CSOs to supply them to their clients [39].

### 4.4. Strengths and Limitations

This study was limited to current smokers and did not include the views of ex-smokers. Focus groups with a range of CSOs and community groups were conducted across the state of Queensland in order to obtain diversity in our sample and to capture a range of views. The sample size and self-selecting nature of the sample should be taken into consideration when generalising these data to other populations. Views on nicotine products are likely to be shaped by the experience and availability of products. Thus, our findings should be interpreted within the context of regulatory and medical context of nicotine products in Queensland, Australia. We did not explore views on other potential harm reduction strategies, such as cutting down, due to the greater uncertainty about the health benefits of this approach, particularly in the absence of alternative sources of nicotine. Finally, the sample size was not large enough to tease out differences according to gender, ethnicity, age or locality. Further studies on the views of people with mental disorders are warranted and could explore the relevance of these factors. More detailed studies on preferences for nicotine products could be used to create decision-tools for clinicians and mental health consumers to aid them in selecting the most appropriate nicotine product.

## 5. Conclusions

Encouraging consumers and clinicians to consider tobacco harm reduction options could help to reduce the disproportionate burden of disease people living with mental disorders experience. Achieving this goal will require communication that challenges black and white thinking, in which smoking cigarettes or quitting all nicotine are viewed as the only legitimate options; this type of perfectionism serves to support the continuance of smoking in practice. Reframing nicotine products as a viable alternative to smoking outside a “quitting“ context could make them more acceptable to these smokers. Local health organisations and CSOs could help consumers by subsidising trials of multiple nicotine products to find the most acceptable alternative to smoking that reduces health harms. Policy and regulatory changes could provide healthier choices for consumers who wish to stop smoking but are unable or unwilling to stop using nicotine.

## Figures and Tables

**Table 1:** Definitions and summary statistics on nicotine product use and willingness to try.

Nicotine Product	Product Description	Product Availability	Willingness to Try		Have Used Previously	
			*n*	%	*n*	%
Tank style vaporiser	Nicotine (tank) vaporisers are an electronic vaporising device that resembles a large fountain pen. It is a similar technology to, but more powerful than, an e-cigarette. The user must regularly refill the “tank“ with liquid containing nicotine.	Devices and nicotine-free liquids for nicotine vaporisers are available legally in most Australian jurisdictions or with nicotine by blackmarket.	19	65.5%	N/A ^1^	
E-cigarette	E-cigarettes are a small disposable electronic vaporising device that delivers nicotine in a visible mist. Visually resembles a cigarette.	Devices and nicotine-free liquids for nicotine vaporisers are available legally in most Australian jurisdictions or with nicotine by blackmarket.	19	65.5%	N/A ^1^	
Nicotine aerosol inhaler	A stick device resembling a cigarette that is filled with an aerosol containing nicotine from a canister similar to an asthma inhaler. Aerosol is released from the stick when user draws on mouthpiece.	Not available for public sale.	17	58.6%	N/A	
Mouth spray	Liquid containing nicotine that is sprayed directly into the mouth.	For sale in Australia.	16	55.2%	5	17.2%
Lozenge	Resembles a small mint lozenge but contains nicotine. Slowly dissolves in the mouth.	For sale in Australia.	14	48.3%	8	27.6%
Inhalator	Cylindrical plastic device (shorter and wider than a cigarette) into which a small cartridge containing nicotine is placed. Nicotine is released as a vapour on inhaling from the mouthpiece.	For sale in Australia.	13	44.8%	8	27.6%
Dissolvable strips	A dissolvable clear film containing nicotine which is placed on the tongue and pressed to the roof of the mouth where it dissolves to release nicotine.	For sale in Australia.	13	44.8%	3	10.3%
Snus	A small pouch of tobacco resembling a teabag that is placed in the mouth, usually between the top lip and gums.	Not for sale in Australia.	12	41.4%	N/A	
Patch	Adhesive film containing nicotine that is applied to skin. Nicotine absorbs through the skin.	For sale in Australia, government subsidised if prescribed.	12	41.4%	16	55.2%
Gum	Resembles regular chewing gum, but contains nicotine. Nicotine is absorbed through the lining of the mouth.	For sale in Australia.	12	41.4%	13	44.8%
NRT ever used ^2^			N/A		16	55.2%

List is ranked in order from most to least popular nicotine product, in terms of willingness to try. NRT—Nicotine Replacement Therapy; N/A—Products not widely available in Australia at time of focus group; ^1^ Two participants provided write-in responses, indicating that they had used e-cigarettes. Further participants in focus groups indicated they had used e-cigarettes.; ^2^ Based on self-reported answer to the question “Have you used NRT previously?”

**Table 2:** Consumer reported views on nicotine products.

Product	Strengths	Weaknesses
Vaping devices (tank vaporisers and e-cigarettes)	Mimics positive elements of smoking for those who enjoy the habit and hit Limited negative elements of smoking No negative effects on others Some preferred bigger hit from nicotine vaporiser in comparison with e-cigarette Different preferences for appearance of device Visual dissimilarity of tank vaporisers an advantage for some	Cost and accessibility, including legality Concerns about long term health impacts and quality control Substitution of habit viewed negatively by those who want to stop habit of smoking Environmental impacts of disposable e-cigarettes E-cigarettes may not provide a big enough hit Potential for difficulties with police associated with the tank-style vaporiser, if misidentified as drug paraphernalia Tank vaporiser visually dissimilar to cigarettes, compared with e-cigarettes, and visually unappealing for some Bulkiness of tank vaporisers unacceptable for some
Nicotine Aerosol Inhaler	Mimics look of cigarette	Less similar look to cigarettes than e-cigarettes Substitution of habit viewed negatively by those who want to stop habit of smoking Cost
Mouth spray	Acceptability dependent on taste	Scepticism of effectiveness due to mode of delivery of nicotine Negative side-effects e.g., nausea
Nicotine inhalator	Mimics some elements of smoking Possibility of use in places where smoking bans are in place No taste	No smoke (or vapour) produced (cf. vaping devices) Greater ability to smoke where bans are in place, may add to rather than replace smoking Visually unappealing, embarrassing to be seen using (looks like a tampon)
Dissolvable oral strips		Not strong enough
Snus	Recognised as less harmful than cigarette smoking Possibility of use in places where smoking bans are in place	Mode of ingestion had mixed acceptability Infeasible for participants with dental and oral prostheses
Patch	Belief they work for people with the “right mindset”	Considered ineffective Negative side-effects e.g., unpleasant dreams and allergic reactions to patch Does not help replace habit of smoking Belief that efficacy is reduced overtime as tolerance builds
Gum	Perceived as effective	Considered ineffective Negative side-effects. e.g., nausea Unpalatable Difficulty in using as directed (gum hardens when “parked”) Infeasible for participants with dental and oral prostheses
Lozenges	Favourable kinetics Described as effective in conjunction to the use of e-cigarettes Individual preferences for different lozenge types (taste) Perceived as cost-effective	Unpalatable Perceived as expensive Slow or ineffective in reducing cravings Negative side effects e.g., mouth ulcers Described as “more addictive” than cigarettes

**Table 3:** Consumer reported facilitators and barriers to using Nicotine Products as a long term substitute for smoking.

Theme	Facilitators to Long Term Use of Nicotine Products	Barriers and Risks to Long Term Use of Nicotine Products
Swapping one addiction for another	Understanding and agreement (implicitly or explicitly) with harm reduction principles with respect to nicotine	Addiction or habit seen as inherently “bad” Risk of substituting smoking for another addiction (e.g., alcohol or food)
What it means to quit	Nicotine products that effectively mimic smoking means that one can continue their lifestyle with reduced health risks	View that quitting is part of a transformation to a healthy lifestyle that does not include smoking or other nicotine use
Appraisal of health risks	Nicotine products recognised as having fewer health risks	Comfort with health risks of continued smoking, health arguments minimally persuasive Higher standards of health and risk applied to alternatives to smoking cigarettes
Consumer Appeal	Creating consumer demand through marketing viewed as an effective means of encouraging switching Recognised aesthetic and sensory similarities between nicotine products and cigarettes Pleasant taste and favourable sensory experience	Desire for transformative change that does not include continuing the performance of smoking, discourages use of nicotine products that mimic cigarettes Use of devices that are mistaken for smoking may attract the stigma of smoking
Cost	Free trials and subsidies on products would incentivise switching	If costs are perceived as similar to cigarettes, cigarette smoking is preferred Unwillingness to waste money on trialling nicotine products with uncertain effectiveness

## Supplementary Materials

The following are available online at www.mdpi.com/1660-4601/13/11/1166/s1, “Focus Group Topic Guide” which provides the focus group discussion guide for this study.
