# Supplementary material for: Views and Preferences for Nicotine Products as an Alternative to Smoking: A Focus Group Study of People Living with Mental Disorders
Source: Int J Environ Res Public Health. 2016 Nov 23;13(11):1166. doi: 10.3390/ijerph13111166 (PMC5129376; doi:10.3390/ijerph13111166)
Supplement: Supplementary file 1 [file ijerph-13-01166-s001.pdf]

Supplementary Materials: Views and Preferences for Nicotine Products as an Alternative to Smoking: A Focus Group Study of People Living with Mental Disorders

Carla Meurk, Pauline Ford, Ratika Sharma, Lisa Fitzgerald and Coral Gartner

1. Topic Guide—Semi-Structured

1.1. Smoking

What do you enjoy about smoking?
What don't you enjoy about smoking?
Do you consider yourself addicted to smoking?
How do you know when someone is addicted to smoking?
Why do people become addicted to smoking?
For those of you who are thinking about quitting, what are some reasons you would like quit?
For those of you not thinking about quitting, what would make you want to quit?
How might quitting be different for smokers with a mental health disorder than for other people?
What methods have you tried to help you quit smoking in the past? How did it go?

1.2. Quitting and Availability and Adequacy of Quitting Services

What do you think is the best way to quit smoking?
Do you think people need support to quit smoking?
Do you know of any services people could use to quit?
What advice would you give someone else if they were wanting to quit?
Do you think there is enough support to quit? Why/why not?
What else do you think should be available to help smokers quit?
Switching from cigarettes to a nicotine product (long-term substitution): If there was a product that you found acceptable to use and that was likely to have less health risks than smoking, would you be interested in using that product instead of smoking as a long-term replacement for cigarettes?
Tell me why swapping smoking (long term substitution) for another product would be attractive or not to you as an option.

1.3. Products

1.3.1. Nicotine Replacement Therapies: Patches, Lozenges, Gum, Strips, Mouth Spray

Show selection of currently available NRT products short identification of each of these products.
Do you think any of these products would be useful for quitting smoking? Why/why not?
Do you think any of these products could be useful as a long term replacement for cigarettes? Why/Why not? Which products?
Do you think smokers would be interested in using any of these products as a long term substitute for cigarettes? Why/why not?
What would encourage people to use these types of products?

1.3.2. Novel Products + Inhalator

Show one product at a time Inhalator, snus, e cigs (2 or 3 types), including explanation of action.

1.3.3. Questions on Each Product

Did anyone not know about this product before today?
Do you think this would be a useful aid to quitting smoking? Why/why not?
Do you think this would be a useful long term replacement for cigarettes? Why/why not?
Do you think people would be interested in using this product? Why/why not?
What would encourage people to use it?
What do you think would stop people from using this product?
Do you think there could be any problems with using this product? What type of problems?
What if anything would make it difficult to stay on this product for 12 weeks or longer?

1.4. Final Questions

Out of these products you say today, which if any would you prefer to use and why?
What would be the most you would be willing to pay for this product for the amount equivalent to a pack of 25 cigarettes?
Do you think any of these products might be misused?

© 2016 by the authors; licensee MDPI, Basel, Switzerland. This article is an open access article distributed under the terms and conditions of the Creative Commons by Attribution (CC-BY) license (http://creativecommons.org/licenses/by/4.0/).
